# *Rickettsia parkeri* Rickettsiosis in Kidney Transplant Recipient, North Carolina, USA, 2023

**DOI:** 10.3201/eid3007.240217

**Published:** 2024-07

**Authors:** Gautam M. Phadke, Kiran Gajurel, Jennifer Kasten, Marlene DeLeon-Carnes, Carmen Ramos, Sandor E. Karpathy, Arlyn N. Gleaton, Sydney N. Adams, Pallavi D. Annambhotla, Sridhar V. Basavaraju, Carl Williams, Christopher D. Paddock

**Affiliations:** Metrolina Nephrology Associates, Charlotte, North Carolina, USA (G. Phadke);; Carolinas Medical Center, Atrium Health, Charlotte (G. Phadke, K. Gajurel);; Centers for Disease Control and Prevention, Atlanta, Georgia, USA (J. Kasten, M. DeLeon-Carnes, C. Ramos, S. Karpathy, A.N. Gleaton, S.N. Adams, P.D. Annambhotla, S.V. Basavaraju, C.D. Paddock);; Oak Ridge Institute for Science and Education, Oak Ridge, Tennessee, USA (S.N. Adams);; North Carolina Department of Health and Human Services, Raleigh, North Carolina, USA (C. Williams)

**Keywords:** Rickettsia, bacteria, vector-borne infections, Rickettsia parkeri, rickettsiosis, kidney transplant, Amblyomma maculatum, Gulf Coast tick, ticks, North Carolina, United States

## Abstract

Spotted fever rickettsiosis is rarely observed in solid organ transplant recipients, and all previously reported cases have been associated with tick bite months to years after transplantation. We describe a kidney transplant recipient in North Carolina, USA, who had a moderately severe *Rickettsia parkeri* infection develop during the immediate posttransplant period.

Spotted fever rickettsiosis in solid organ transplant recipients is rarely described, and all reports document disease acquired months to years after the transplant, after recognized tick bites or exposures to tick-infested habitats (*1*–*4*). In the United States, *Rickettsia parkeri* rickettsiosis is a tickborne infection transmitted by the Gulf Coast tick (*Amblyomma maculatum*) that results in a disease similar to but milder than Rocky Mountain spotted fever. Human infection with *R. parkeri* was first described in 2004 (*5*). We report a case of *R. parkeri* rickettsiosis in a kidney transplant recipient in North Carolina, USA, during the immediate posttransplant period.

## The Case

**Figure 1:**
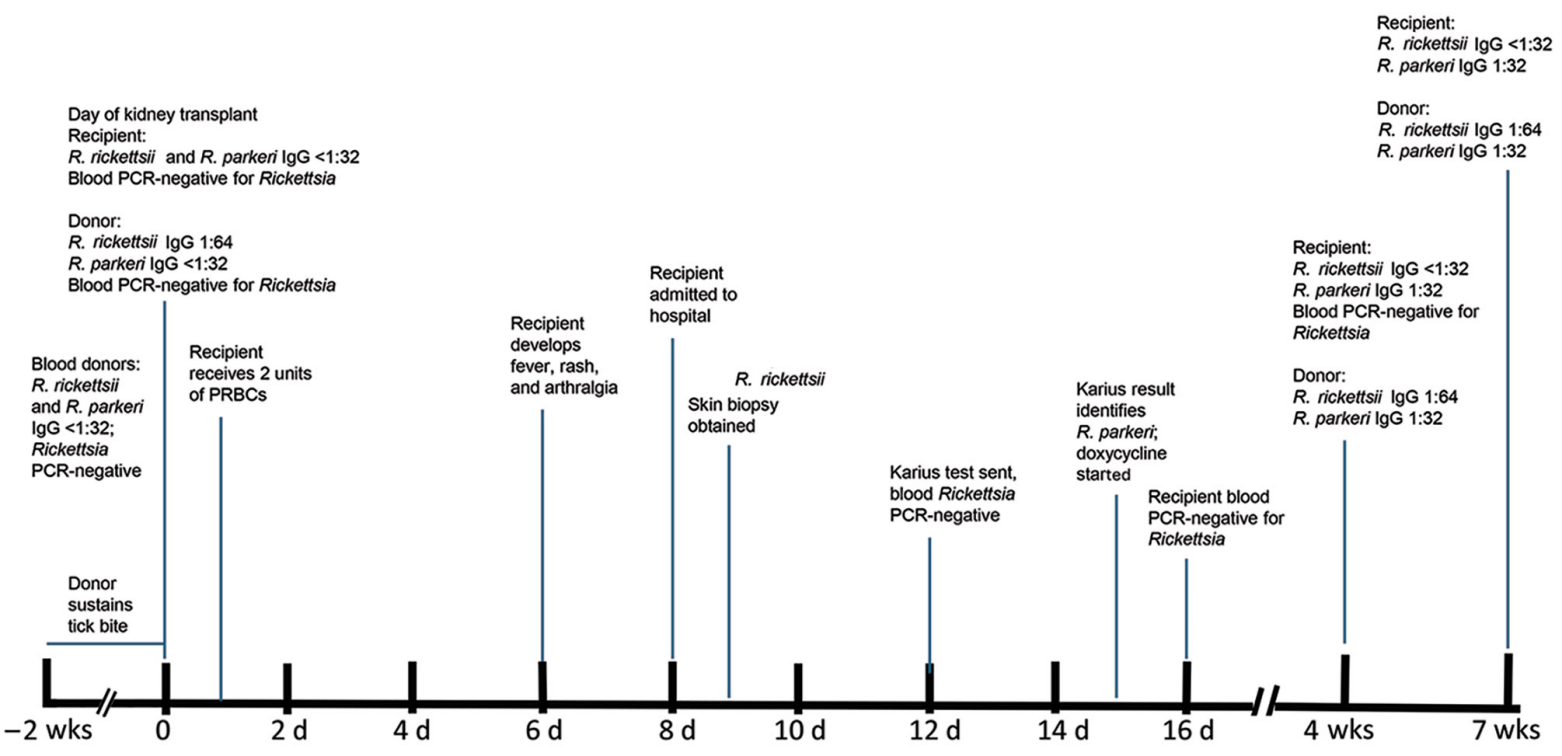
Transmission and testing timeline for case of *Rickettsia parkeri* rickettsiosis in a kidney transplant receipient, North Carolina, USA, 2023. Pretransplant samples were tested retrospectively to determine possible transmission risk from 2 blood donors and the kidney donor. PRBCs, packed red blood cells.

**Figure 2:**
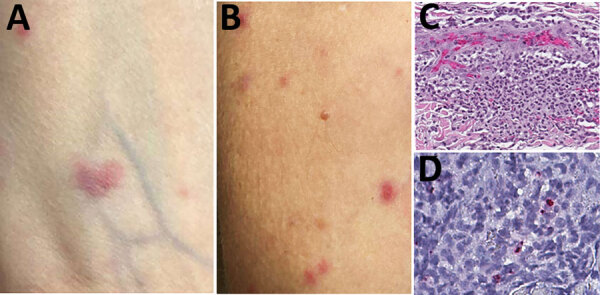
Skin lesions and testing results for a kidney transplant receipient diagnosed with *Rickettsia parkeri* rickettsiosis, North Carolina, USA, 2023. A, B) Sparse maculopapular rash involving forearms. Lesions ranged from 0.2 to 3 cm in greatest dimension and were tender and erythematous. C) Histopathologic appearance of rash lesion demonstrating perivascular collections of mixed inflammatory cell infiltrates in the mid-dermis comprising predominantly neutrophils and macrophages. Hematoxylin and eosin stain; original magnification ×50. D) Immunohistochemical detection of antigens of *R. parkeri* (red) in dermal inflammatory cell infiltrates. Immunoalkaline phosphatase with naphthol-fast red and hematoxylin counterstain; original magnification ×100.

In July 2023, a 56-year-old woman in central North Carolina who had end-stage renal disease from autosomal dominant Alport syndrome received a living unrelated kidney transplant from a 52-year-old woman, also a resident of central North Carolina (Figure 1). The recipient received alemtuzumab and methylprednisolone for induction immunosuppression and tacrolimus and mycophenolate for maintenance immunosuppression. She received 2 units of packed red blood cells from 2 blood donors, from central North Carolina and western South Carolina, on the first day after transplantation. Five days later, fever developed (101°F), along with a diffuse rash involving her chest and upper and lower extremities and arthralgia involving her ankles, knees, and hips. Seven days after transplantation, arthralgia extended to her elbows, wrists, and metacarpophalangeal joints, and additional rash lesions appeared on her extremities and chest. She was readmitted the next day with pain in the left knee and left elbow. A tender maculopapular rash, comprising ≈30 lesions of 0.2–3 cm in greatest dimension, was identified on her chest and extremities (Figure 2, panels A, B). No eschars were identified.

At admission, laboratory results were notable for elevated erythrocyte sedimentation rate (26 mm/h; reference <20 mm/h) and C-reactive protein (3.2 mg/dL; reference <0.5 mg/dL). Peripheral leukocyte and platelet counts remained within reference limits during her hospitalization. Because of a history of gout, empiric methylprednisolone (100 mg/d for 3 d) was initiated. The patient subsequently had intermittent low-grade fever to 100.9°F, mild elevations in hepatic transaminases (peak aspartate aminotransferase 75 U/L [reference 13–39 U/L], peak alanine aminotransferase 109 U/L [reference 7–52 U/L]), additional rash lesions on her extremities and chest, and 2-mm tender ulcerated lesions on her inner lip. Nine days after transplantation, a biopsy from a rash lesion showed multifocal perivascular neutrophilic infiltrates. A plasma specimen was obtained 12 days after transplantation and evaluated by a microbial cell-free DNA test (Karius, https://kariusdx.com) that identified *R.* *parkeri* (88 DNA molecules/μL, reference <10 DNA molecules/μL). Therapy with oral doxycycline (100 mg 2×/d) was initiated ≈2 weeks after transplantation. Her arthralgias improved within 2 days, and rash resolved within 5 days. Approximately 3 weeks after completing a 14-day course of doxycycline, she reported mild arthralgia involving hand, ankle and hip joints, and a few macular lesions recurred on her extremities. Doxycycline was administered for an additional 2 weeks, and complete resolution of disease ensued.

The transplant recipient denied recent tick bite or exposure to tick-infested habitats during the 2 weeks before transplantation and remained indoors from the time of hospital discharge until readmission. Neither blood donor reported a tick bite in the 2 weeks preceding their donations in late June and early July or an illness during the 2 weeks after donation. The blood collection establishment identified no similar illnesses in other recipients who received other blood products from those donors. The kidney donor recalled a bite from a large brown tick with white markings ≈2 weeks before transplantation; however, the donor had no illness in the subsequent weeks.

The Centers for Disease Control and Prevention tested residual pretransplant and posttransplant serum samples from the kidney donor and the recipient and samples from each blood donor at the time of donation by using indirect immunofluorescence antibody assays to detect IgG reactive with *R. rickettsii* and *R. parkeri* antigens (*5*). Titers >64 were considered positive. DNA extracted from residual whole blood, serum, or plasma samples from the recipient, kidney donor, and blood donors was tested by using a real-time PCR targeting a segment of the 23S rRNA gene of *Rickettsia* spp. (*6*) and by an *R. parkeri*–specific real-time PCR (*7*). The Centers for Disease Control and Prevention also evaluated the skin biopsy specimen, stained by an immunoalkaline phosphatase technique for multiple species of spotted fever group *Rickettsia*, including *R. parkeri*; DNA was extracted and evaluated by a multiplex real-time PCR to detect *R.* *parkeri* (*8*). Cycle threshold values <40 were considered positive for each PCR.

The 4 serum specimens from the kidney recipient collected 2 weeks pretransplant through 7 weeks posttransplant showed titers <32 against both antigens (Figure 1). Serum specimens obtained from the kidney donor on the day of transplant through 7 weeks posttransplant revealed a stationary titer of 64 to antigens of *R. rickettsii* and titers <32 to those of *R. parkeri*. Aliquots obtained from both samples of residual transfused blood revealed titers <32 to *R. parkeri* and *R. rickettsii* antigens*.* No *R. parkeri* DNA was detected in pretransplant or posttransplant whole blood, serum, or plasma specimens from the recipient or from the organ or blood donors. 

Histologic evaluation of the skin biopsy specimen revealed small vessel vasculitis with thrombi, perivascular inflammatory cell infiltrates comprising neutrophils, lymphocytes, and macrophages; and periadnexal fibrinoid necrosis (Figure 2, panel C). An immunohistochemical stain for spotted fever group *Rickettsia* spp. identified rickettsial antigens in the inflammatory cell infiltrates (Figure 2, panel D), and real-time PCR detected *R. parkeri* DNA in the tissue.

## Conclusions

We identified *R. parkeri* rickettsiosis in a kidney transplant recipient in the immediate posttransplant period. The organ donor, 1 of the blood donors, and the recipient resided in the Piedmont region of North Carolina, where *R. parkeri*–infected Gulf Coast ticks have been identified repeatedly (*9*,*10*). The mode of acquisition of *R. parkeri* remains undetermined, although evidence indicates this infection was possibly acquired through transplantation (*11*), including absence of tick bite or exposure to tick-infested habitat by the recipient during the 2 weeks before transplantation; absence of an inoculation eschar in the recipient; and documented tick bite of the donor by an adult tick morphologically compatible with *A. maculatum* 2 weeks before organ procurement. Neither an illness compatible with a rickettsioisis nor a seroconversion to antigens of spotted fever group *Rickettsia* were identified for the donor; nonetheless, recent surveys suggest that up to 30%–40% of adult *A. maculatum* ticks are infected with *R. parkeri* in the Piedmont region of North Carolina where the donor resided (*9**,**10*). The donor reported frequent tick bites during her lifetime, suggesting the possibility of an asymptomatic infection after repeated past exposures to *R. parker*i (*12*). Transfusion-associated transmission of *R. rickettsii*, the agent of Rocky Mountain spotted fever, has been described (*13*); however, we believe that transmission route was less likely because of the composite histories, negative serologic and molecular test results of residual blood from each transfused unit, and absence of illness compatible with rickettsiosis in the blood donors or other recipients of blood products from the blood donors. Lack of a serologic response in the recipient was likely related to an intensive immunosuppresive regimen that included alemtuzumab during the postoperative period.

This case highlights the utility of increasingly sensitive and commercially available molecular methods to detect otherwise unsuspected or difficult-to-diagnose infectious diseases, including rickettsioses, in solid organ transplant recipients (*14*). Although tickborne pathogens constitute a small proportion of infections transmitted by solid organ transplants (*15*), enhanced awareness of their potential occurrence, coupled with increasingly sensitive methods of pathogen-diagnostic laboratory detection, will reveal additional cases of rickettsial diseases in this patient cohort.
